# Three New Pigment Protein Tyrosine Phosphatases Inhibitors from the Insect Parasite Fungus *Cordyceps gracilioides*: Terreusinone A, Pinophilin C and Cryptosporioptide A

**DOI:** 10.3390/molecules20045825

**Published:** 2015-04-02

**Authors:** Pei-Yao Wei, Lin-Xia Liu, Ting Liu, Chuan Chen, Du-Qiang Luo, Bao-Zhong Shi

**Affiliations:** 1 College of Life Science, Key Laboratory of Medicinal Chemistry and Molecular Diagnosis of Ministry of Education, Hebei University, Baoding 071002, China; E-Mails: weipeiyao_218@163.com (P.-Y.W.); liulinxia.hi@163.com (L.-X.L.); liuting61800@163.com (T.L.); hyperchuan@aliyun.com (C.C.); 2 Biotechnology Center of Hebei Province, Hebei University, Baoding 071002, China; E-Mail: shibaozhong111@163.com

**Keywords:** protein tyrosine phosphatases inhibitors, *Cordyceps gracilioides*, terreusinone A, pinophilin C, cryptosporioptide A

## Abstract

Three new pigment compounds—terreusinone A (**1**), pinophilin C (**2**) and cryptosporioptide A (**3**) were isolated from a solid culture of *Cordyceps gracilioides*. The structures of these compounds were determined by extensive spectroscopic analysis including HRESIMS, 1D- and 2D-NMR. The structure of terreusinone A (**1**) was further confirmed by single-crystal X-ray crystallographic diffraction analysis. In an *in vitro* activity assay, **1**, **2** and **3** exhibited high inhibitory activity against PTP1B, SHP2, CDC25B, LAR and SHP1. Terreusinone A (**1**) inhibited PTP1B, SHP2, CDC25B, LAR and SHP1 enzyme with IC_50_ values 12.5, >50, 4.1, 10.6, 5.6 µg/mL, respectively; pinophilin C (**2**) with IC_50_ values 6.8, 8.0, 4.5, 4.7, 3.4 µg/mL, respectively; and cryptosporioptide A (**3**) with IC_50_ values 7.3, 5.7, 7.6, >50, 4.9 µg/mL, respectively.

## 1. Introduction

**Figure 1:**
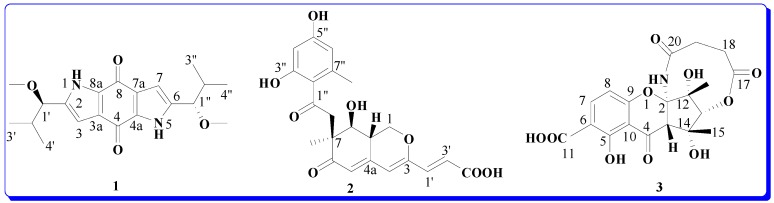
The structures of compounds **1**–**3**.

*Cordyceps* is a genus of ascomycete fungi that includes about 400 species, which are parasitic mainly on insects and other arthropods [1]. *Cordyceps* are rich sources of novel biologically active substances with diverse structural architectures [2], such as the antimalarial erythrostominones [3], antimalarial cordypyridones [4], antitrypanosomal cardinalisamides A–C [5] and opaliferi [6]. *Cordyceps gracilioides Kobayasi* was a new species in China and the specimen was collected on a coleopteran from the National Natural Conservation of Guniujiang, Shitai County, Anhui Province in September 2000 [7]. Although there has been considerable investigation on the genus *Cordyceps*, little has been reported about secondary metabolites from *C. gracilioides*. Here we describe the isolation and structural elucidation of the three new compounds—terreusinone A (**1**), pinophilin C (**2**) and cryptosporioptide A (**3**, Figure 1)—from *C. gracilioides* and the inhibitory activities against the enzymes PTP1B, SHP2, CDC25B, LAR and SHP1.

## 2. Results and Discussion

**Figure 2:**
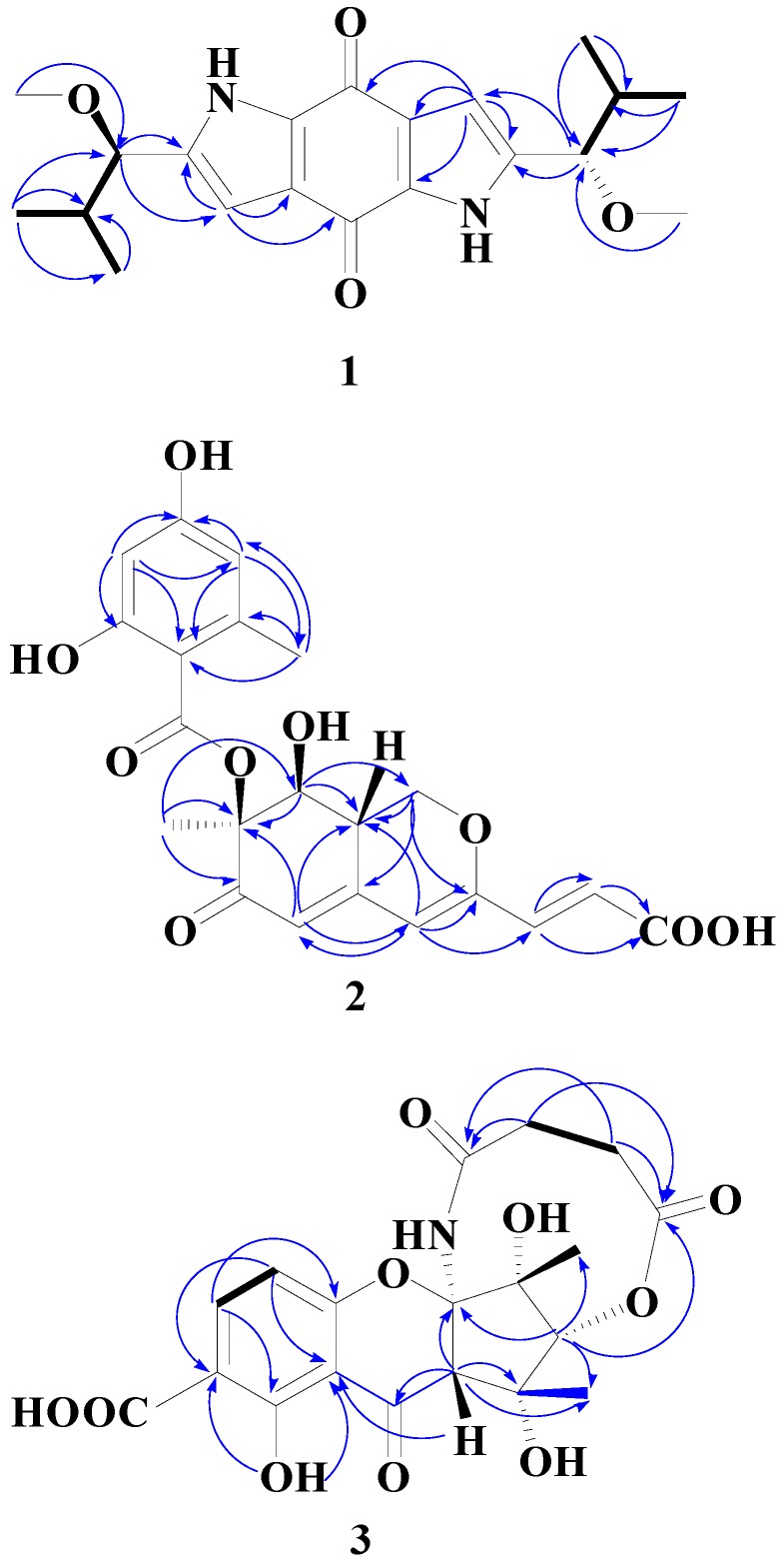
^1^H-^1^H COSY correlations (bold lines) and selected HMBC (^1^H→^13^C) (single lines) correlations of **1**–**3**.

**Figure 3:**
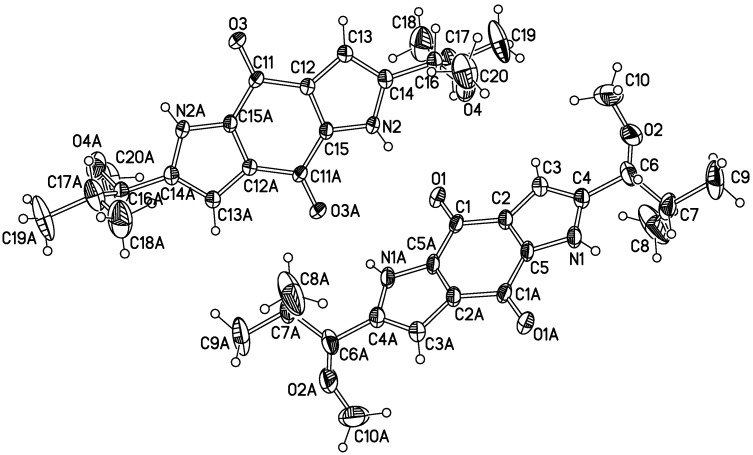
X-ray crystal structure of compound **1**.

Compound **1** was obtained as red crystals (from MeOH). The high-resolution EIMS exhibited a molecular ion peak at *m/z* 358.1893, corresponding to the molecular formula of C_20_H_26_N_2_O_4_ (calcd. 358.1880), indicating nine degrees of unsaturation. The IR absorption spectrum of **1** showed bands characteristic of benzoquinone (1631 cm^−1^) and pyrrole (3442, 1469 cm^−1^) functionalities. The ^13^C-NMR (DEPT) spectrum of **1** showed only 10 signals: three olefinic quaternary carbons (δ_C_ 139.1, 126.5, 132.7), one olefinic methine carbons (δ_C_ 106.7), two methine carbons (δ_C_ 83.0, 33.4), three methyl carbons (δ_C_ 18.4, 18.0, 56.8) and one carbonyl group (δ_C_ 175.2) for one half of the molecule, suggesting that **1** is a high symmetry dimer. The ^1^H- and ^13^C-NMR and HMBC data (Table 1) of **1** were similar to that of terreusinone, which suggested the compound possesses the same dipyrrolobenzoquinone skeleton [8]. The key difference between **1** and terreusinone was that the hydroxyl in terreusinone was replaced by a methoxy in **1**. The structure of **1** was further confirmed by long range correlations measured in an HMBC experiment (Figure 2). ROESY spectral data which showed the correlations of H-1' to H-3, H-1 to H-OCH_3_ indicative of their *trans* orientations, respectively. The relative configuration was further confirmed by X-ray diffraction (Figure 3). Thus, compound **1** was identified as 2,6-bis(1-methoxy-2-methylpropyl) pyrrolo[2,3-f]indole-4,8(1*H*,5*H*)-dione as shoen in Figure 1, and it was named terreusinone A.

**Table 1:** NMR Spectroscopic Data for Compound **1** (Terreusinone A) in CD_3_OD-*d*_6_.

Position	δ_H_^a^ (*J* in Hz)	δ_C_^b^, mult	HMBC(H→C#)
2 (6)		139.1 (C)	
3 (7)	6.42 (s)	106.7 (CH)	1' (1''), 8a(4a), 3a(7a), 2(6), 4(8)
3a (7a)		126.5 (C)	
4 (8)		175.2 (C)	
4a (8a)		132.7 (C)	
1' (1'')	3.86 (d, *J =* 7.4)	83.0 (CH)	2(6),3(7),2' (2''),4' (4'') 1' (1'')-OCH_3_, 3' (3'')
2' (2'')	2.03 (m)	33.4 (CH)	2(6), 1' (1''), 3' (3''), 4' (4'')
3' (3'')	1.00 (d, *J =* 6.6)	18.4 (CH_3_)	1' (1''), 2' (2''), 4' (4'')
4' (4'')	0.80 (d, *J =* 6.6)	18.0 (CH_3_)	1' (1''), 2' (2''), 3' (3'')
1' (1'')-OCH_3_	3.26 (s)	56.8 (CH_3_)	1' (1'')

^a^ ^1^H-NMR were recorded in CD_3_OD-*d*_6_ at 600 MHz; ^b^ ^13^C-NMR were recorded in CD_3_OD-*d*_6_ at 150 MHz.

The molecular formula of **2** was deduced from the HRESI-MS [M+H]^+^ peak at *m/z* 417.1180 (calcd. for C_21_H_21_O_9_ 417.1188), indicating 12 degrees of unsaturation. The IR spectrum indicated the presence of a hydroxy group (3427 cm^−1^), a conjugated ester (1703 cm^−1^), and a conjugated carbonyl (1636 cm^−1^). As shown in Table 2, the ^1^H- and ^13^C-NMR spectra suggested that compound **2** had a similar structures to pinophilin A [9] and differed only in C-3' of the side chain, whereby the methyl group in pinophilin A was replaced by a carboxylic acid group in **2** [δ_C_ 168.0 (COOH, C-3')]. The azaphilone skeleton was further confirmed by long range correlations measured in an HMBC experiment (Figure 2). The relative configuration of compound **2** was determined by ^1^H-^1^H coupling and NOESY correlations. The *anti* relationship of H-8/H-8a was deduced from the coupling constants (*J*_8, 8a_ = 9.6 Hz). The *syn* relationship of H-8a/7-*O*-benzoyl group was determined from the NOESY correlations between 7-CH_3_ and H-8. The CD spectrum of compound **2** exhibited Cotton effects due to the interaction between the benzoate and conjugated trienone chromophores at 326 and 301 nm (Δε −15.1 and 5.0), indicating the *S* configuration [10] at C-7. Thus, compound **2** was identified as (*E*)-3-((7*S*,8*S*,8a*S*)-7-((2,4-dihydroxy-6-methylbenzoyl)oxy)-8-hydroxyl-7-methyl-6-oxo-6,7,8,8a-tetra-hydro-1*H*-isochromen-3-yl)acrylic acid as shown in Figure 1, and it was named pinophilin C.

Compound **3** was obtained as a yellow solid. The high-resolution EIMS exhibited a molecular ion peak at *m/z* 421.1012 corresponding to the molecular formula of C_19_H_19_NO_10_ (calcd. 421.1009), indicating 11 degrees of unsaturation. The IR spectrum displayed absorption bands at 3440 (OH), 3030, 2931 (CH), 1745 (C=O) and 1591, 1562 (C=C) cm^−1^. The ^1^H-NMR spectrum of **3** (Table 3) showed two *ortho*-coupled doublets in the aromatic region at δ_H_ 7.40 (1H, *J* = 8.4 Hz, H-7) and 6.42 (1H, *J* = 8.4 Hz, H-8), which are attributed to a tetrasubstituted benzene ring.

It also showed δ_H_ 5.65 (1H, s, H-13), 3.42 (1H, s, H-3), 2.65 (2H, m, H-18), 2.66 (2H, m, H-18), 1.56 (3H, s, H-15), 1.65 (3H, s, H-16). The most downfield signals appeared at δ_H_ 14.03 (1H, s, NH) and 11.75 (1H, s, OH). The unusual downfield shift of the amide hydrogen could be due to an anisotropic effect of oxygen of the chromone moiety. The ^13^C-NMR (DEPT) spectrum of **3** (Table 3) showed signals corresponding to 19 signals: two methyls (δ_C_ 28.4, 18.1), two methylenes (δ_C_ 28.8, 28.8), four methines (δ_C_ 140.5, 108.2, 72.5, 55.8) and 11 quaternary carbons (δ_C_ 187.8, 177.2, 171.2, 169.8, 159.0, 157.1, 117.1, 106.0, 104.3, 78.9, 58.7). The ^1^H- and ^13^C-NMR and HMBC experiment results (Table 3) of **3** were similar to those of cryptosporioptide [11]. The distinct differences between **3** and cryptosporioptide were that a methoxy group [δ_C_ 52.5 (OCH3)] in cryptosporioptide was replaced by an OH in **3** and one methylene (δ_C_ 41.1, C-18) in cryptosporioptide was replaced by two methylenes [δ_C_ 28.8, 28.8 (C-18, 19)] in **3**. The polyketide skeleton was further confirmed by long range correlations measured in an HMBC experiment (Figure 2). The *syn* relationship of 15-Me/16-Me/H-3/H-13 was determined from NOESY correlations between 15-Me, 16-Me, H-3, and H-13. The absolute configuration of C-2, 3, 12, 13, 14 in **3** was further determined by comparing the circular dichroism (CD) and [α]_D_ spectra with cryptosporioptide (see Supporting Information). The (CD) and [α]_D_ of **3** were very similar to cryptosporioptide, therefore, we may assign the absolute configuration of compound 3 as (+)-(2*R*, 3*S*, 12*R*, 13*R*, 14*R*)-5,12,14-trihydroxy-12,14-dimethyl-4,17,20-trioxo-3,7,8, 13,18,19-octahydro-21*H*-2,13-methanochromeno[3,2-*d*][1,6]oxazecine-11-carboxylic acid, named cryptosporioptide A (3) as shown in Figure 1.

**Table 2:** NMR Spectroscopic Data for Compound **2** (Pinophilin C) in CD_3_OD-*d*_6_.

Position	δ_H_^a^ (*J* in Hz)	δ_C_^b^, Mult	HMBC(H→C#)
1	4.81(dd, *J =* 10.7, 5.3) 3.81(dd, *J =* 13.6, 10.7)	68.8 (CH_2_)	8a, 4a, 3
3		157.0 (C)	
4	6.04 (s)	109.6 (CH)	8a, 5, 3, 1'
4a		149.2 (C)	
5	5.94 (d, *J =* 2.0)	119.5 (CH)	8a, 7, 4,
6		194.2 (C)	
7		84.7 (C)	
7-Me	1.67 (s)	17.0 (CH_3_)	
8	3.62 (d, *J =* 9.6)	74.2 (CH)	7-Me, 8a, 1, 7
8a	3.25 (dddd, *J =* 13.6, 9.6, 5.3, 2.0)	37.5 (CH)	8, 4a
1'	7.05 (d, *J =* 15.4)	137.1 (CH)	4, 2', 3, 3'
2'	6.33 (d, *J =* 15.4)	122.3 (CH)	1', 3, 3'
3'		168.0 (COOH )	
1''		169.8 (C)	
2'		105.1 (C )	
3''		163.9 (C)	
4'	6.14 (d, *J =* 2.4)	100.4 (CH)	2'', 6'', 5'', 3''
5''		162.4 (C)	
6''	6.20 (d, *J =* 2.4)	111.0 (CH)	7''-Me, 2'', 4'', 5''
7''		143.1 (C)	
7''-Me		23.0 (CH_3_)	

^a^ ^1^H-NMR were recorded in CD_3_OD-*d*_6_ at 600 MHz; ^b^ ^13^C-NMR were recorded in CD_3_OD-*d*_6_ at 150 MHz.

Compounds **1**–**3** were examined for their inhibitory activity against the enzymes PTP1B, SHP2, CDC25B, LAR and SHP1 using an *in vitro* assay. The results showed that **1** and **2** have significant inhibitory activity against CDC25B and SHP1, with IC_50_ values of 4.1, 3.4 µg/mL, respectively (Table 4).

**Table 3:** NMR Spectroscopic Data for Compound **3** (Cryptosporioptide A) in CDCl_3_-*d*_6_.

Position	δ_H_^a^ (*J* in Hz)	δ_C_^b^, mult	HMBC(H→C#)
2	-	104.3 (C)	
3	3.42 (s)	55.8 (CH)	2, 4, 11, 14, 15
4	-	187.8 (C)	
5	-	159.0 (C)	
6	-	117.1 (C)	
7	7.40 (d, *J* = 8.4)	140.5 (CH)	5, 9, 10
8	6.42 (d, *J* = 8.4)	108.2 (CH)	4, 6, 9, 10
9	-	157.1 (C)	
10	-	106.0 (C)	
11	-	169.8 (COOH)	
12	-	78.9 (C)	
13	5.65 (s)	72.5 (CH)	2, 3, 4, 12, 14, 15, 16, 17
14	-	58.7 (C)	
15	1.56 (s)	18.1 (CH_3_)	3, 13, 14,
16	1.65 (s)	28.4 (CH_3_)	2, 12, 13
17	-	171.2 (C)	
18	2.65 (m)	28.8 (CH_2_)	17
19	2.66 (m)	28.8 (CH_2_)	20
20	-	177.2 (C)	
5-OH	11.75 (s)	-	5, 6, 10
N–H	14.03 (s)	-	

^a^ ^1^H-NMR were recorded in CDCl_3_-*d*_6_ at 600 MHz; ^b^ ^13^C-NMR were recorded in CDCl_3_-*d*_6_ at 150 MHz.

**Table 4:** The activity of enzyme inhibition of compounds **1**–**3**.

Compounds	IC_50_ Values (µg/mL)				
	PTP1B	SHP2	CDC25B	LAR	SHP1
**1**	12.5	>50	4.1	10.6	5.6
**2**	6.8	8.0	4.5	4.7	3.4
**3**	7.3	5.7	7.6	>50	4.9
**Sodium orthovanadate (positive control)**	38.3	122.2	0.93	163.0	4.3

## 3. Experimental Section

### 3.1. General Information

The ^1^H- and ^13^C-NMR spectra were recorded on a Bruker AM-600 spectrometer (Faellanden, Switzerland) at 600 and 150.9 MHz using TMS as internal standard. Optical rotations were obtained on a Perkin-Elmer 341 spectropolarimeter (Milpitas, CA, USA). IR spectra were recorded on a Perkin-Elmer 577 spectrometer (Waltham, MA, USA). HRESIMS data were measured on a Bruker FT-ICR-MS mass spectrometer (Billerica, MA, USA). UV Spectra were recorded on a UV-210 spectrometer (Kyoto, Japan). Column chromatography (CC): silica gel (200~300 mesh, Yantai Zhi Fu Chemical Co. Ltd., Yantai, China), TLC: silica gel GF254 plates (Yantai Zhi Fu Chemical Co. Ltd.) and Sephadex LH-20 gel (25~100 µm, GE Healthcare Co. Ltd., Uppsala, Sweden).

### 3.2. Fungal Material and Cultivation Conditions

The *Cordyceps gracilioides Kobayasi* was isolated from Coleoptera larvae collected in Guniujiang (Anhui Province, China), and assigned the accession number ACCC47758 in the culture collection at the College of Life Science, Key Laboratory of Medicinal Chemistry and Molecular Diagnosis of Ministry of Education, Hebei University. The fungal strain was cultured on slants of potato dextrose agar (PDA) at 28 °C for 7 days, and then inoculated into a 500 mL Erlenmeyer flask containing 100 mL of PDA medium (20.0 g of glucose, 200.0 g of potato (peeled), 3.0 g of KH_2_PO_4_, 1.5 g of MgSO_4_, 0.1 g of citric acid, and 10.0 mg of thiamin hydrochloride, in 1 L of deionized H_2_O). The final pH of the medium was adjusted to 6.5 before sterilization. After 7 days of incubation at 28 °C on rotary shakers at 150 rpm, 25 mL of culture liquid were transferred as seed into a 500 mL Erlenmeyer flask containing 200.0 g rice medium, and the fermentation was carried out in an incubator for 40 days.

### 3.3. Extraction and Isolation

The culture broth (30 L) was extracted three times with ethyl acetate (30 L, soaking each time for 2 days), and the organic layer was concentrated *in vacuo* to yield a brown oily residue (70 g). This residue was subjected to silica gel column chromatography (CC) with elution using a gradient of petroleum ether/EtOAc (100:0, 98:2, 95:5, 90:10, 60:10, 30:10, 10:10 (v/v)) to obtain seven fractions 1–7. Fraction 5 (150 mg) eluted with petroleum ether/EtOAc (60:10) (v/v) was repeatedly purified by CC (silica gel; petroleum ether/EtOAc 10:1 (v/v)) and Sephadex LH-20 chromatography (CHCl_3_/MeOH, 1:1 (v/v)) to afford **2** (20 mg) and **3** (30 mg) as yellow amorphous powders. Fraction 6 (100 mg) eluted with petroleum ether/EtOAc (30:10) (v/v) was repeatedly purified by CC (silica gel; petroleum ether/EtOAc 5:1 (v/v), Sephadex LH-20 chromatography (CHCl_3_/MeOH, 1:1 (v/v)) and Sephadex LH-20 (methanol) to give **1** (15 mg) as red crystals.

### 3.4. Analytical Data

#### 3.4.1. Terreusinone A (**1**)

Red crystals. HREIMS *m/z* 358.1893 (calcd. for C_20_H_26_N_2_O_4_ 358.1880); [α]_D_^21.5^ = −7° (c, 0.001, MeOH); UV (MeOH) λ_max_ (logε): 357 (3.93), 287 (4.10), 250 (4.58) nm; IR (KBr)_vmax_: 3442, 3125, 2960, 1631, 1555, 1469, 1384, 1228, 1162, 1088, 993, 752, 670 cm^−1^; ^1^H- and ^13^C-NMR: Table 1. *X-Ray Crystallographic Analysis of* **1**. Upon crystallization from MeOH by the vapor-diffusion method, red crystals of **1** were obtained. A crystal (0.29 mm × 0.28 mm × 0.20 mm) was separated from the sample and mounted on a glass fiber, and data were collected with a Bruker-SMART-1000-CCD diffractometer and graphite-monochromated MoKa radiation (λ = 0.71073 Å) at 296(2) K. Crystal data: C_20_H_26_N_2_O_4_, M = 358.43, Monoclinic space group: P-1; Unit cell dimensions a = 8.3255(10) Å, b = 9.9479(12) Å, c = 12.4585(15) Å. V = 968.9(2) Å^3^, Z = 2, D = 1.229 mg/m^3^. F (000) = 384, µ = 0.086 mm^−1^.The structure was solved by direct methods with SHELXL-97 and refined by full-matrix least-squares difference Fourier techniques. All non-H-atoms were refined with anisotropic displacement parameters, and all H-atoms were placed in idealized positions and refined as riding atoms with the relative isotropic parameters. Absorption corrections were applied with the Siemens area detector absorption program (SADABS). The 5740 measurements yielded 4139 independent reflections after equivalent data were averaged, and Lorentz and polarization corrections were applied. The final refinement gave R_f_ = 0.1278 and R_w_ = 0.4121 (I > 2s (I)). Crystallographic data for the structure of **1** has been deposited in the Cambridge Crystallographic Data Centre (deposition number: CCDC 1041522). Copies of these data can be obtained, free of charge, on application to the CCDC through http://www.ccdc.cam.ac.uk/conts/retrieving.html (or from the Cambridge Crystallographic Data Centre, 12, Union Road, Cambridge CB2 1EZ, UK; Fax (Internat.): +44-1223/336-033; E-Mail: deposit@ccdc.cam.ac.uk).

#### 3.4.2. Pinophilin C (**2**)

Yellow solid. HRESI-MS [M+H]^+^ *m/z* 417.1180 (calcd. for C_21_H_21_O_9_ 417.1188); [α]_D_^21.6^ = −146° (c, 0.002, MeOH); IR(KBr_)_ ν_max_: 3427, 2962, 2933, 2875, 2374, 1703, 1636, 1589, 1489, 1447, 1382, 1200, 1172, 1103, 995, 616 cm^−1^; UV (MeOH) λ_max_ (logε): 347 (4.36), 266 (4.23), 216 (4.45) nm; ^1^H- and ^13^C-NMR: Table 2.

#### 3.4.3. Cryptosporioptide A (**3**)

Yellow solid. HREIMS *m/z* 421.1012 (calcd.for C_19_H_19_NO_10_ 421.1009); [α]_D_^21.5^ = +20.1° (c, 0.003, MeOH); IR(KBr) _νmax_: 3440, 3030, 2931, 1745, 1591, 1562 cm^−1^; UV (MeOH) λ_max_ (log ε): 388 (3.59), 203 (3.61) nm; ^1^H- and ^13^C-NMR: Table 3.

### 3.5. PTP Assay

Protein tyrosine phosphatases (PTPs) constitute a large family of signaling enzymes that control the cellular levels of protein tyrosine phosphorylation. Several “classical” PTPs are attractive therapeutic targets, including PTP1B for obesity and Type II diabetes, SHP1, and SHP2 and CDC25B for cancer [12]. GST-ΔPTP1B, GST-ΔSHP2, GST-ΔSHP1, GST-ΔLAR and GST-ΔCDC25B fusion proteins which only carry the PTP activity domain were expressed in *Escherichia coli* DH5α and affinity purified with glutathione Sepharose (Amersham Biosciences, Piscataway, NJ, USA). *p*-Nitrophenyl phosphate (pNPP) was used in the enzymatic reactions to determine the intrinsic catalytic activities of the PTPs. All assays using pNPP as the substrate were performed in a buffered reaction medium containing 10 mM of sodium acetate (NaAc), 1 mM of ethylenediaminetetraacetic acid and 1 mM of dithiothreitol at 37 °C, pH 5.5. Sodium orthovanadate was used as the positive control. Every experiment was performed in triplicate. The inhibitory activity measurements of PTPs were performed with different concentrations (0, 2.5, 5, 10, 20, 40 µg mL^−1^) of inhibitor and 20 µg mL^−1^ of PTPs. The reaction mixture was incubated at 37 °C for 15 min and the catalytic activity was detected by monitoring the absorbance at 405 nm immediately after adding 12.5 mM of pNPP. IC_50_ (the concentration of an inhibitor that is required for 50% inhibition of an enzyme) data were derived from the experimental results [13,14,15,16,17].

## 4. Conclusions

In summary we have isolated three new pigments—terreusinone A (**1**), pinophilin C (**2**) and cryptosporioptide A (**3**)—from cultures of the insect fungus *Cordyceps gracilioides*. We also demonstrate for the first time that **1** and **2** have significant inhibitory activity against CDC25B and SHP1.

## Supplementary Materials

Supplementary materials can be assessed at: http://www.mdpi.com/1420-3049/20/04/5825/s1.
