# Supplementary material for: Three New Pigment Protein Tyrosine Phosphatases Inhibitors from the Insect Parasite Fungus Cordyceps gracilioides: Terreusinone A, Pinophilin C and Cryptosporioptide A
Source: Molecules. 2015 Apr 2;20(4):5825–34. doi: 10.3390/molecules20045825 (PMC6272775; doi:10.3390/molecules20045825)
Supplement: Supplementary file 1 [file molecules-20-05825-s001.pdf]

Supplementary Materials

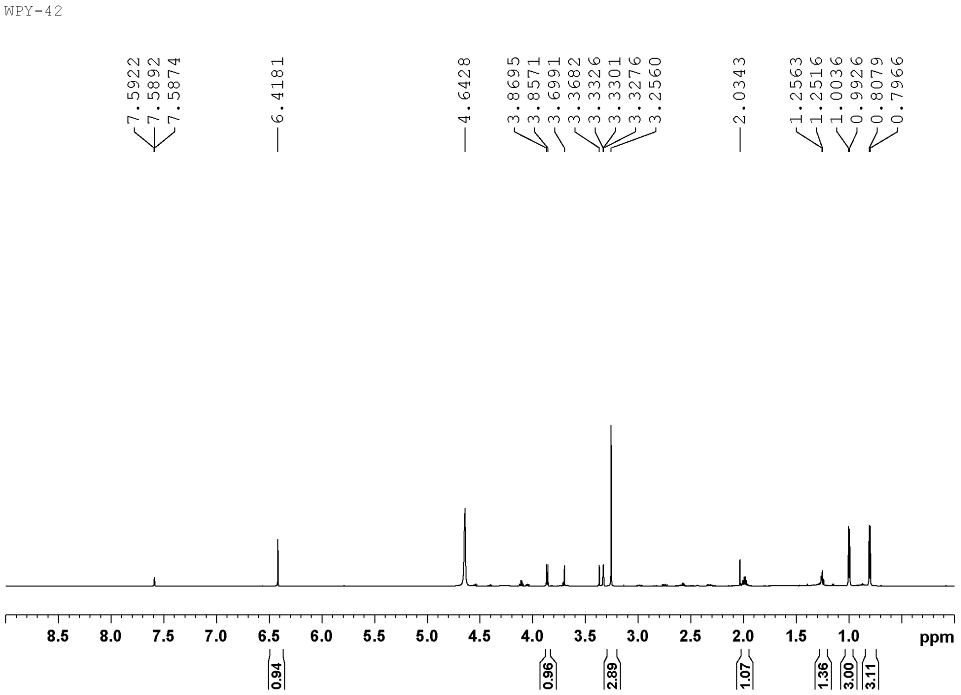

Figure S1. ^{1}H-NMR of **1**.

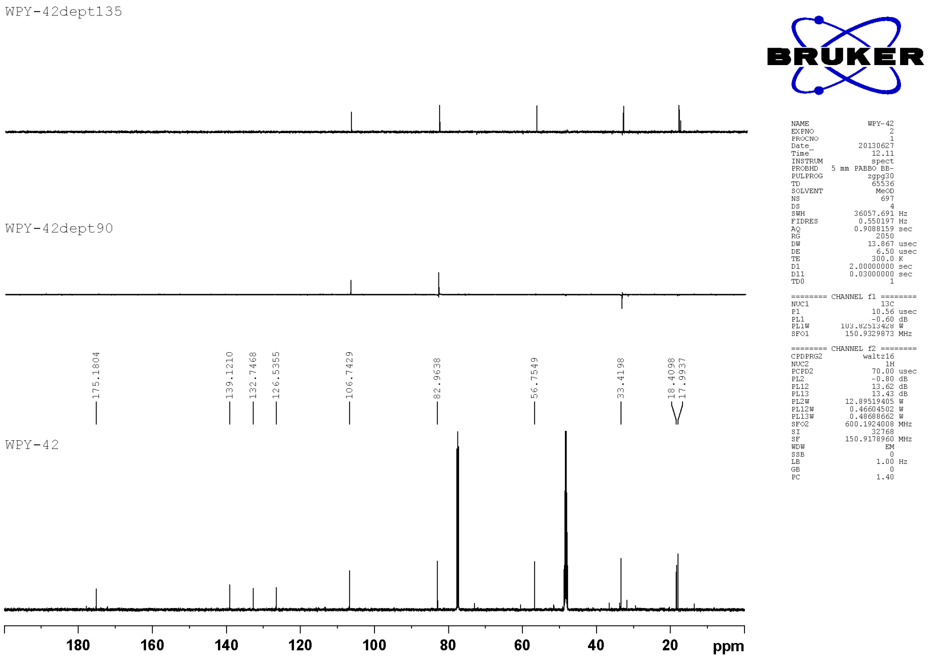

Figure S2. ^{13}C-NMR of **1**.

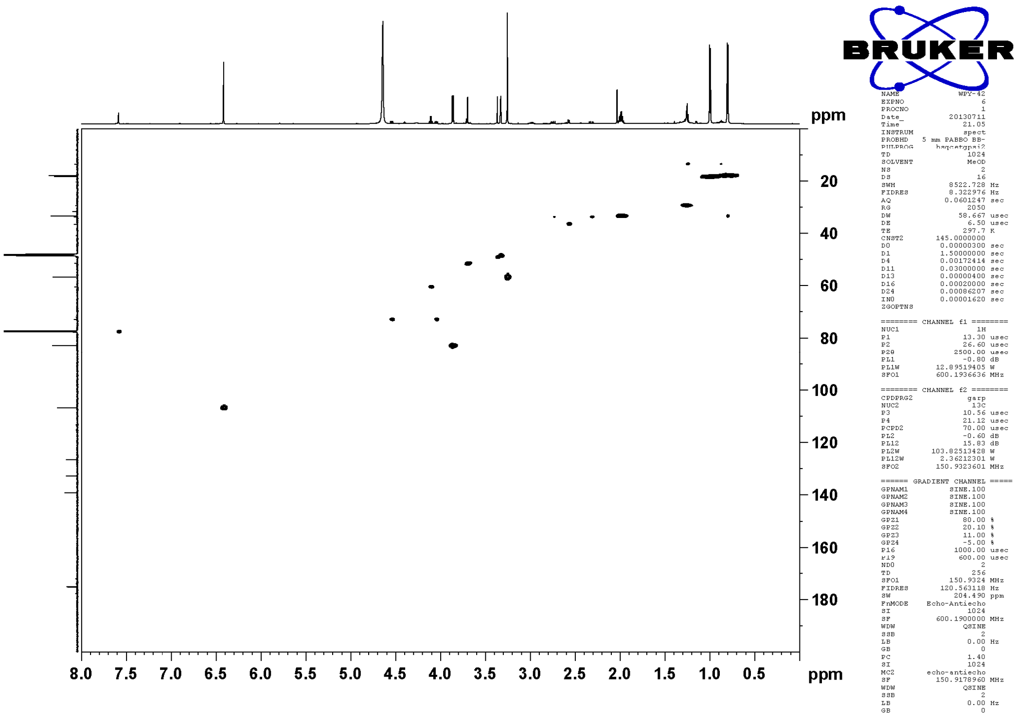

Figure S3. HSQC of **1**.

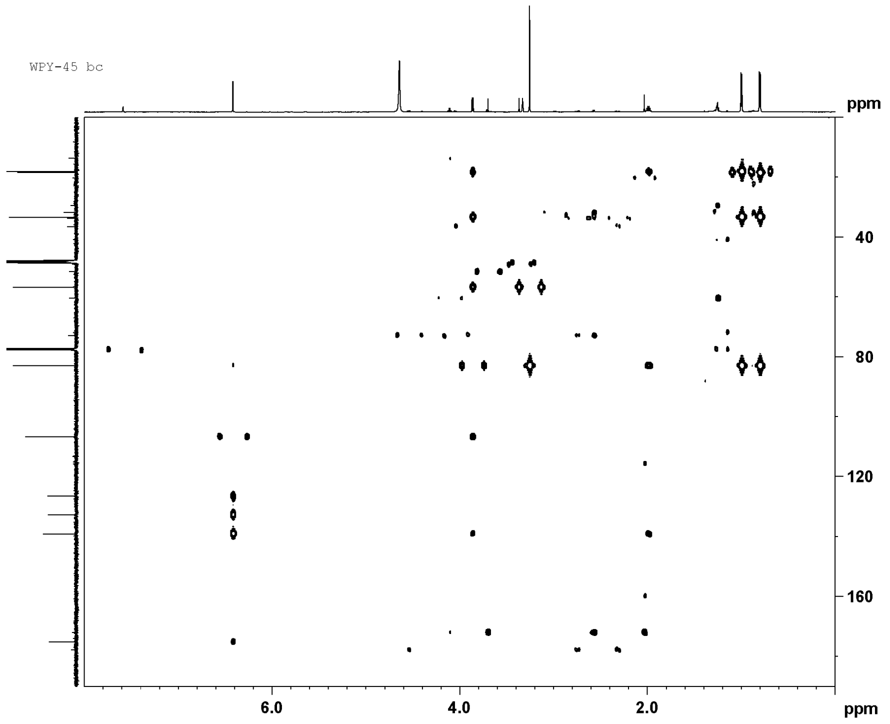

Figure S4. HMBC of **1**.

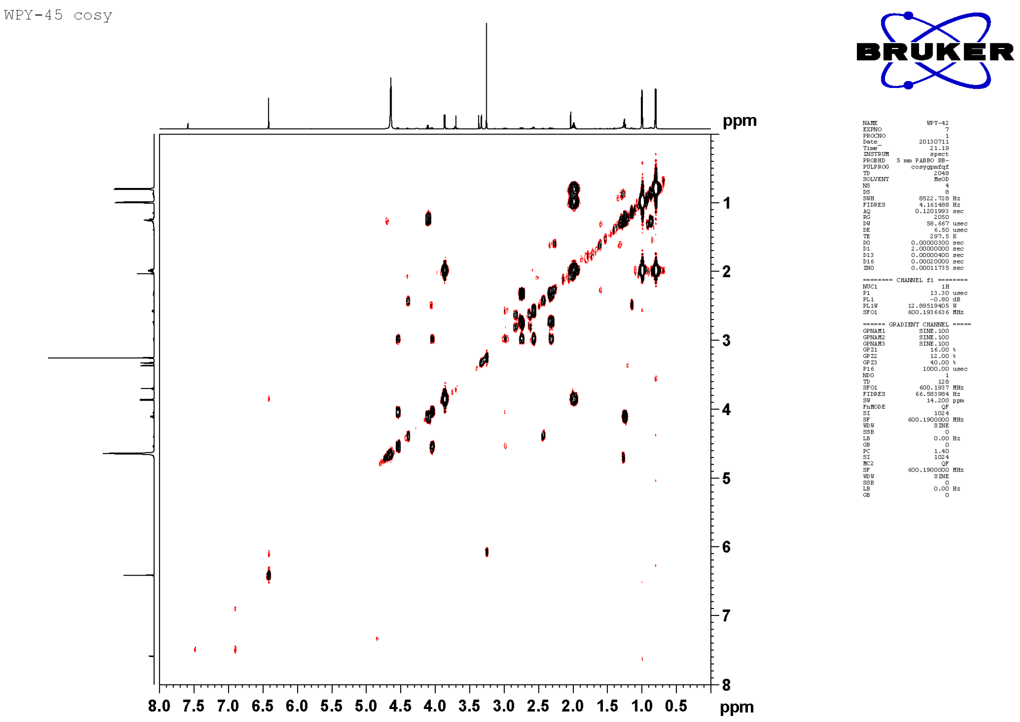

Figure S5. H-H COSYof **1**.

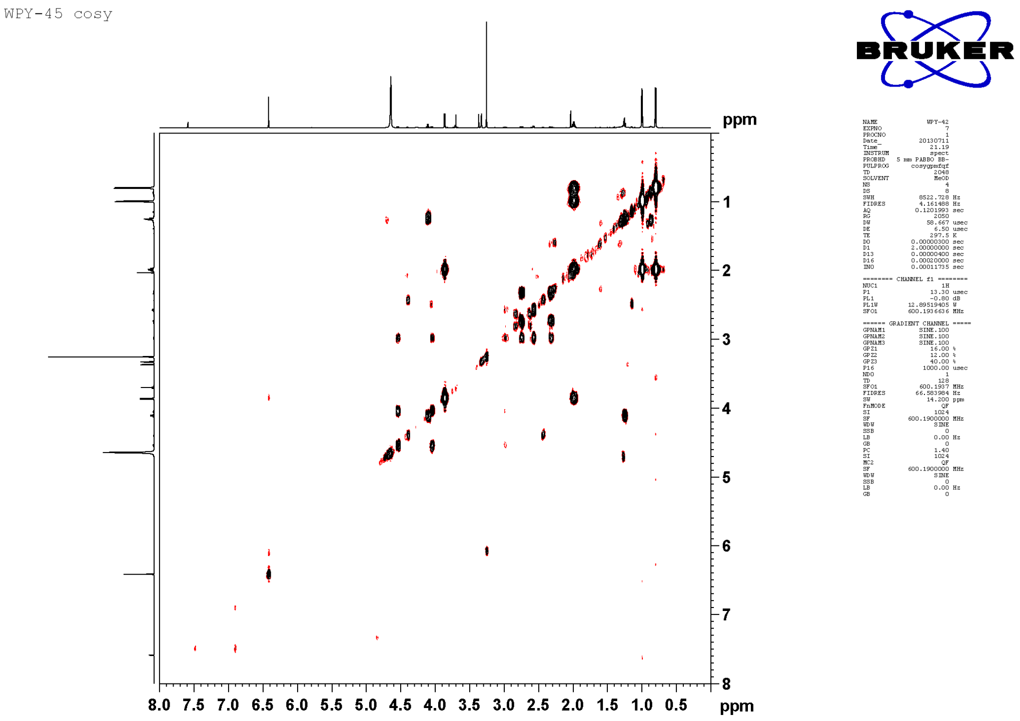

Figure S6. NOESY of **1**.

Elemental Composition Report **Page 1**

Single Mass Analysis
Tolerance = 10.0 PPM / DBE: min = -10.0, max = 120.0
Selected filters: None

Monoisotopic Mass, Odd and Even Electron Ions
178 formula(e) evaluated with 3 results within limits (up to 51 closest results for each mass)
Elements Used:
C: 0-200 H: 0-400 N: 0-4 O: 3-9

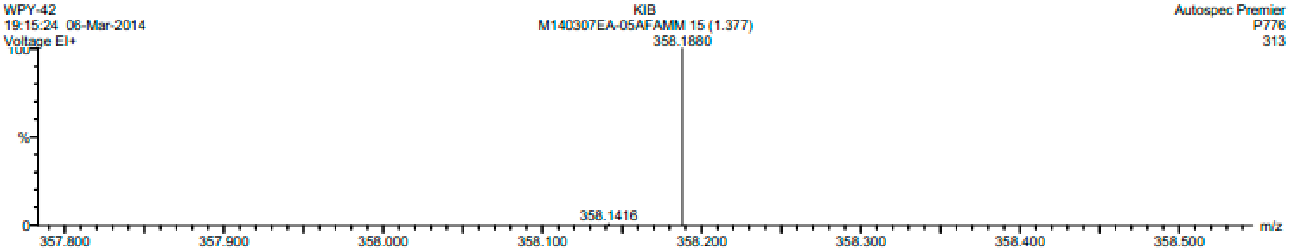

Minimum:				-10.0		
Maximum:		200.0	10.0	120.0		
Mass	Calc. Mass	mDa	PPM	DBE	i-FIT	Formula
358.1880	358.1893	-1.3	-3.6	9.0	5546169.0	C20 H26 N2 O4
	358.1866	1.4	3.9	4.5	5546165.0	C17 H28 N O7
	358.1852	2.8	7.8	5.0	5546163.5	C15 H26 N4 O6

Figure S7. Single Mass Analysis of **1**.

Table S1. Crystal data and structure refinement for **1**_0m.

**Parameters**	**Data**
Identification code	1_0m
Empirical formula	$C_{20}H_{26}N_2O_4$
Formula weight	358.43
Temperature	296(2) K
Wavelength	0.71073 Å
Crystal system	Triclinic
Space group	P-1
Unit cell dimensions	a = 8.3255(10) Å, alpha =96.166(2)°
	b = 9.9479(12) Å, beta =107.299(2)°
	c = 12.4585(15) Å, gamma =96.127(2)°
Volume, Z	968.9(2) Å^3, 2
Density (calculated)	1.229 mg/m^3
Absorption coefficient	0.086 mm^{-1}
F(000)	384
Crystal size	0.29 mm × 0.28 mm × 0.20 mm
θ range for data collection	2.08 to 27.00°
Limiting indices	−10 < h < 10, −12 < k < 9, −14 < l < 15
Reflections collected	5740
Independent reflections	4139 (Rint = 0.0127)
Completeness to θ = 27.00°	97.5%
Absorption correction	Semi-empirical from equivalents
Max. and min. Transmission	0.9831 and 0.9756
Refinement method Full-matrix least-squares on F2	
Data/restraints/parameters	4139/1/250
Goodness-of-fit on F2	1.742
Final R indices [I>2s(I)]	R1 = 0.1278, wR2 = 0.4121
R indices (all data)	R1 = 0.1576, wR2 = 0.4386
Largest diff. peak and hole	1.450 and −1.483 eÅ^{-3}

Table S2. Atomic coordinates [× 104] and equivalent isotropic displacement parameters [A2 × 103] for 1_0m. U(eq) is defined as one third of the trace of the orthogonalized Uij tensor.

	**x**	**y**	**z**	**U(eq)**
N(1)	567(5)	7724(4)	818(4)	51(1)
N(2)	4656(4)	2309(3)	1252(3)	41(1)
O(1)	1699(4)	7724(4)	818(4)	51(1)
O(2)	3662(9)	9403(10)	3072(13)	368(12)
O(3)	8267(3)	-280(3)	116(3)	49(1)
O(4)	5850(6)	5307(4)	2018(4)	90(1)
C(1)	941(5)	4134(4)	821(4)	45(1)
C(2)	1047(5)	5618(4)	1083(4)	47(1)
C(3)	1966(6)	6527(4)	2103(4)	53(1)
C(4)	1618(6)	7813(4)	1893(4)	56(1)
C(5)	180(5)	6387(4)	324(4)	47(1)
C(6)	2304(7)	9139(5)	2662(5)	65(1)
C(7)	942(9)	9910(7)	2899(5)	96(3)
C(8)	-56(14)	9094(13)	3449(12)	208(7)
C(9)	1725(14)	11288(7)	3623(6)	122(3)
C(10)	5190(9)	9118(9)	2999(9)	129(4)
C(11)	6811(5)	-143(4)	74(3)	39(1)
C(12)	6346(5)	1043(4)	655(3)	39(1)
C(13)	7356(5)	2194(4)	1397(4)	48(1)
C(14)	6279(5)	2973(4)	1746(4)	48(1)
C(15)	4664(5)	1142(4)	581(3)	38(1)
C(16)	6666(6)	4297(5)	2533(4)	59(1)
C(17)	6315(11)	4169(7)	3638(5)	91(2)
C(18)	6961(14)	3099(9)	4181(6)	122(3)
C(19)	6915(15)	5576(8)	4421(6)	144(4)
C(20)	6653(11)	5873(7)	1264(6)	103(2)

TableS3. Bond lengths [Å] and angles [°] for 1_0m.

N(1)-C(4)	1.352(6)
N(1)-C(5)	1.365(5)
N(1)-H(2)	0.93(5)
N(2)-C(15)	1.358(4)
N(2)-C(14)	1.367(5)
N(2)-H(1)	0.81(4)
O(1)-C(1)	1.231(5)
O(2)-C(6)	1.083(7)
O(2)-C(10)	1.359(11)
O(3)-C(11)	1.220(4)
O(4)-C(16)	1.381(6)
O(4)-C(20)	1.433(8)
C(1)-C(5)#1	1.456(6)
C(1)-C(2)	1.464(5)
C(2)-C(5)	1.367(6)
C(2)-C(3)	1.430(6)
C(3)-C(4)	1.376(6)
C(3)-H(3)	0.9300
C(4)-C(6)	1.493(6)
C(5)-C(1)#1	1.456(6)
C(6)-C(7)	1.515(6)
C(6)-H(6)	0.9800
C(7)-C(8)	1.460(14)
C(7)-C(9)	1.520(9)
C(7)-H(7)	0.9800
C(8)-H(8A)	0.9600
C(8)-H(8B)	0.9600
C(8)-H(8C)	0.9600
C(9)-H(9A)	0.9600
C(9)-H(9B)	0.9600
C(9)-H(9C)	0.9600
C(10)-H(10A)	0.9600
C(10)-H(10B)	0.9600
C(10)-H(10C)	0.9600
C(11)-C(15)#2	1.459(5)
C(11)-C(12)	1.465(5)
C(12)-C(15)	1.391(5)
C(12)-C(13)	1.407(5)
C(13)-C(14)	1.379(5)
C(13)-H(13)	0.9300
C(14)-C(16)	1.497(6)
C(15)-C(11)#2	1.459(5)
C(16)-C(17)	1.506(8)
C(16)-H(16)	0.9800
C(17)-C(18)	1.393(10)
C(17)-C(19)	1.558(8)

C(17)-H(17)	0.9800
C(18)-H(18A)	0.9600
C(18)-H(18B)	0.9600
C(18)-H(18C)	0.9600
C(19)-H(19A)	0.9600
C(19)-H(19B)	0.9600
C(19)-H(19C)	0.9600
C(20)-H(20A)	0.9600
C(20)-H(20B)	0.9600
C(20)-H(20C)	0.9600
C(4)-N(1)-C(5)	109.3(4)
C(4)-N(1)-H(2)	128(3)
C(5)-N(1)-H(2)	122(3)
C(15)-N(2)-C(14)	110.0(3)
C(15)-N(2)-H(1)	120(3)
C(14)-N(2)-H(1)	130(3)
C(6)-O(2)-C(10)	142.8(9)
C(16)-O(4)-C(20)	112.6(5)
O(1)-C(1)-C(5)#1	124.0(4)
O(1)-C(1)-C(2)	124.3(4)
C(5)#1-C(1)-C(2)	111.7(3)
C(5)-C(2)-C(3)	107.2(4)
C(5)-C(2)-C(1)	122.7(4)
C(3)-C(2)-C(1)	130.1(4)
C(4)-C(3)-C(2)	106.1(4)
C(4)-C(3)-H(3)	126.9
C(2)-C(3)-H(3)	126.9
N(1)-C(4)-C(3)	108.9(4)
N(1)-C(4)-C(6)	122.9(4)
C(3)-C(4)-C(6)	128.1(5)
N(1)-C(5)-C(2)	108.3(4)
N(1)-C(5)-C(1)#1	126.1(4)
C(2)-C(5)-C(1)#1	125.6(4)
O(2)-C(6)-C(4)	119.6(5)
O(2)-C(6)-C(7)	126.2(6)
C(4)-C(6)-C(7)	113.8(5)
O(2)-C(6)-H(6)	92.3
C(4)-C(6)-H(6)	92.3
C(7)-C(6)-H(6)	92.3
C(8)-C(7)-C(6)	110.2(7)
C(8)-C(7)-C(9)	110.8(6)
C(6)-C(7)-C(9)	110.8(6)
C(8)-C(7)-H(7)	108.3
C(6)-C(7)-H(7)	108.3
C(9)-C(7)-H(7)	108.3
C(7)-C(8)-H(8A)	109.5
C(7)-C(8)-H(8B)	109.5

H(8A)-C(8)-H(8B)	109.5
C(7)-C(8)-H(8C)	109.5
H(8A)-C(8)-H(8C)	109.5
H(8B)-C(8)-H(8C)	109.5
C(7)-C(9)-H(9A)	109.5
C(7)-C(9)-H(9B)	109.5
H(9A)-C(9)-H(9B)	109.5
C(7)-C(9)-H(9C)	109.5
H(9A)-C(9)-H(9C)	109.5
H(9B)-C(9)-H(9C)	109.5
O(2)-C(10)-H(10A)	109.5
O(2)-C(10)-H(10B)	109.5
H(10A)-C(10)-H(10B)	109.5
O(2)-C(10)-H(10C)	109.5
H(10A)-C(10)-H(10C)	109.5
H(10B)-C(10)-H(10C)	109.5
O(3)-C(11)-C(15)#2	123.3(3)
O(3)-C(11)-C(12)	124.1(4)
C(15)#2-C(11)-C(12)	112.6(3)
C(15)-C(12)-C(13)	107.2(3)
C(15)-C(12)-C(11)	121.6(3)
C(13)-C(12)-C(11)	131.1(3)
C(14)-C(13)-C(12)	107.5(4)
C(14)-C(13)-H(13)	126.2
C(12)-C(13)-H(13)	126.2
N(2)-C(14)-C(13)	107.7(3)
N(2)-C(14)-C(16)	122.1(4)
C(13)-C(14)-C(16)	130.2(4)
N(2)-C(15)-C(12)	107.6(3)
N(2)-C(15)-C(11)#2	126.6(3)
C(12)-C(15)-C(11)#2	125.8(3)
O(4)-C(16)-C(14)	112.2(4)
O(4)-C(16)-C(17)	110.4(5)
C(14)-C(16)-C(17)	113.7(4)
O(4)-C(16)-H(16)	106.7
C(14)-C(16)-H(16)	106.7
C(17)-C(16)-H(16)	106.7
C(18)-C(17)-C(16)	115.1(6)
C(18)-C(17)-C(19)	112.5(6)
C(16)-C(17)-C(19)	108.8(6)
C(18)-C(17)-H(17)	106.6
C(16)-C(17)-H(17)	106.6
C(19)-C(17)-H(17)	106.6
C(17)-C(18)-H(18A)	109.5
C(17)-C(18)-H(18B)	109.5
H(18A)-C(18)-H(18B)	109.5
C(17)-C(18)-H(18C)	109.5

H(18A)-C(18)-H(18C)	109.5
H(18B)-C(18)-H(18C)	109.5
C(17)-C(19)-H(19A)	109.5
C(17)-C(19)-H(19B)	109.5
H(19A)-C(19)-H(19B)	109.5
C(17)-C(19)-H(19C)	109.5
H(19A)-C(19)-H(19C)	109.5
H(19B)-C(19)-H(19C)	109.5
O(4)-C(20)-H(20A)	109.5
O(4)-C(20)-H(20B)	109.5
H(20A)-C(20)-H(20B)	109.5
O(4)-C(20)-H(20C)	109.5
H(20A)-C(20)-H(20C)	109.5
H(20B)-C(20)-H(20C)	109.5

Symmetry transformations used to generate equivalent atoms: #1 −x, −y+1, −z; #2 −x+1, −y, −z.

Table S4. Anisotropic displacement parameters [A2 × 103] for 1_0m. The anisotropic displacement factor exponent takes the form: $-2p^2$[(ha*)2U11 + ... + 2hka*b*U12].

	U11	U22	U33	U23	U13	U12
N(1)	55(2)	30(2)	69(2)	−5(2)	25(2)	8(2)
N(2)	40(2)	37(2)	48(2)	−4(1)	17(1)	12(1)
O(1)	59(2)	43(2)	69(2)	6(1)	22(2)	23(2)
O(2)	85(4)	212(9)	600(20)	−304(13)	−96(8)	63(5)
O(3)	33(1)	48(2)	65(2)	−2(1)	15(1)	13(1)
O(4)	106(3)	57(2)	116(4)	3(2)	48(3)	23(2)
C(1)	42(2)	33(2)	64(3)	2(2)	22(2)	14(2)
C(2)	48(2)	33(2)	61(2)	−4(2)	21(2)	8(2)
C(3)	53(2)	42(2)	60(3)	−5(2)	14(2)	10(2)
C(4)	50(2)	41(2)	74(3)	−10(2)	21(2)	8(2)
C(5)	46(2)	29(2)	69(3)	−7(2)	26(2)	8(2)
C(6)	74(3)	41(2)	77(3)	−13(2)	33(3)	−7(2)
C(7)	125(6)	77(4)	64(3)	−25(3)	−6(3)	58(4)
C(8)	152(8)	184(11)	277(14)	−157(10)	154(9)	−83(8)
C(9)	229(10)	62(4)	69(4)	−13(3)	37(5)	46(5)
C(10)	68(4)	96(6)	183(9)	25(6)	−20(5)	9(4)
C(11)	34(2)	37(2)	49(2)	2(2)	15(2)	12(2)
C(12)	36(2)	37(2)	43(2)	−2(2)	12(2)	8(2)
C(13)	38(2)	43(2)	58(2)	−10(2)	14(2)	5(2)
C(14)	48(2)	39(2)	54(2)	−7(2)	17(2)	7(2)
C(15)	40(2)	31(2)	45(2)	−2(2)	17(2)	7(2)
C(16)	58(3)	44(2)	70(3)	−13(2)	21(2)	7(2)
C(17)	135(6)	71(4)	61(3)	−14(3)	35(4)	3(4)
C(18)	195(9)	111(6)	66(4)	4(4)	42(5)	50(6)
C(19)	267(12)	72(4)	85(5)	−38(4)	76(6)	−20(6)
C(20)	149(7)	66(4)	106(5)	20(4)	61(5)	−6(4)

Table S5. Hydrogen coordinates (× 104) and isotropic displacement parameters (A2 × 103) for 1_0m.

	**x**	**y**	**z**	**U(eq)**
H(3)	2658	6296	2772	64
H(6)	2343	9631	2028	78
H(7)	180	10070	2171	115
H(8A)	257	8193	3449	312
H(8B)	−1244	9035	3041	312
H(8C)	163	9518	4217	312
H(9A)	2307	11153	4385	183
H(9B)	845	11835	3631	183
H(9C)	2518	11746	3311	183
H(10A)	5531	9659	2489	193
H(10B)	5094	8166	2719	193
H(10C)	6025	9328	3738	193
H(13)	8535	2394	1613	58
H(16)	7890	4602	2723	71
H(17)	5077	3988	3459	109
H(18A)	6554	2256	3668	183
H(18B)	6593	3042	4838	183
H(18C)	8181	3261	4412	183
H(19A)	6633	5521	5110	216
H(19B)	6358	6265	4036	216
H(19C)	8124	5809	4600	216
H(20A)	7744	6378	1696	155
H(20B)	5956	6471	848	155
H(20C)	6797	5147	742	155
H(1)	3780(50)	2500(40)	1340(30)	25(9)
H(2)	260(60)	8430(60)	400(40)	55(13)

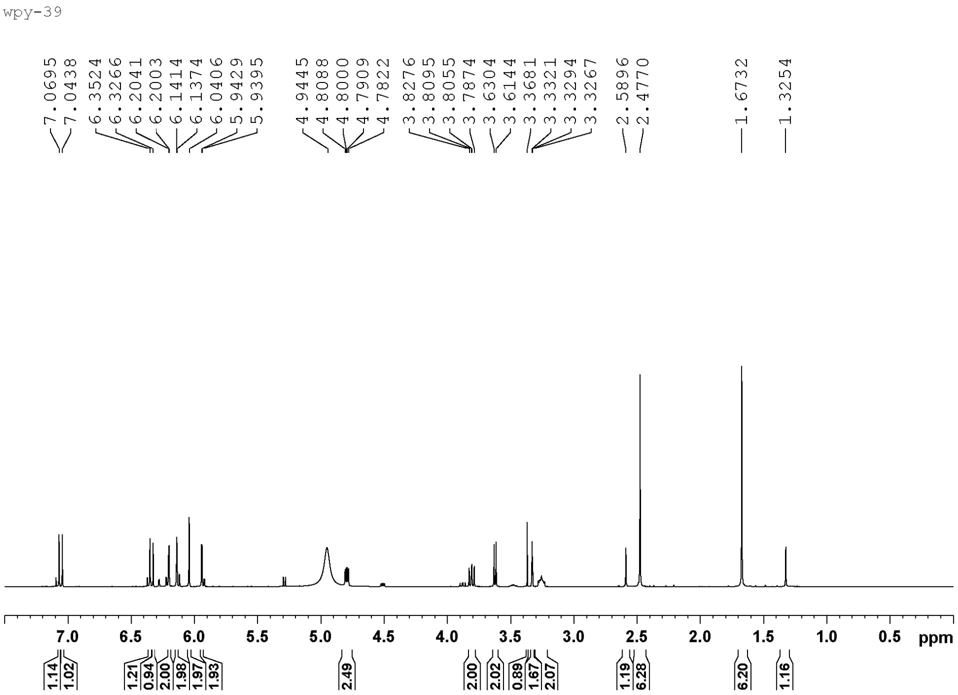

Figure S8. ^{1}H−NMR of **2**.

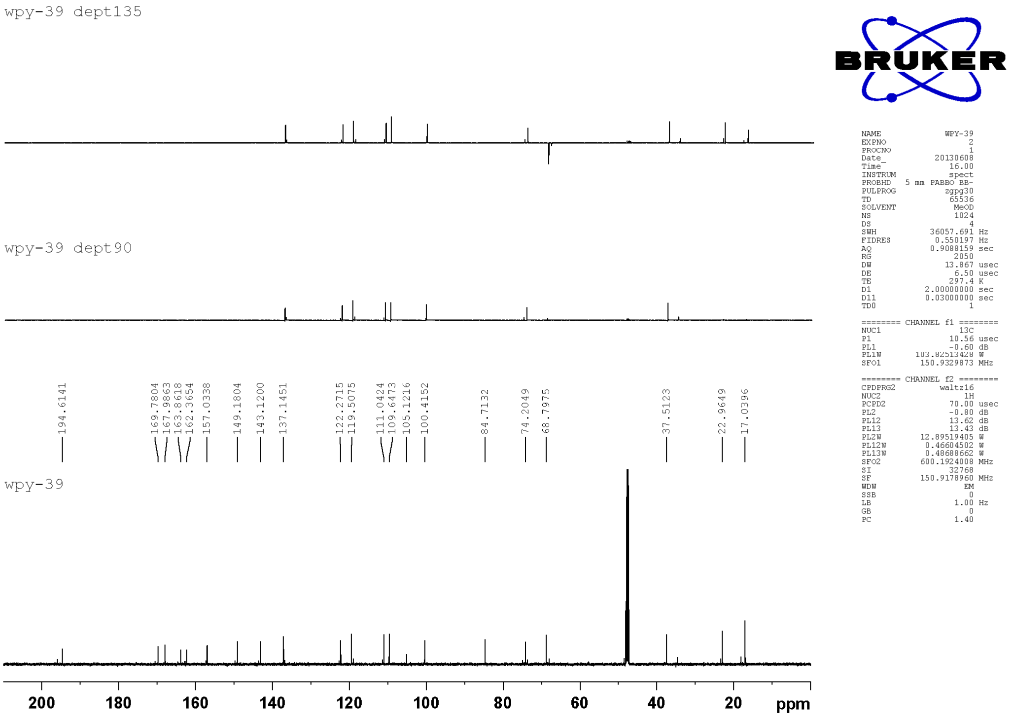

Figure S9. ^{13}C−NMR of **2**.

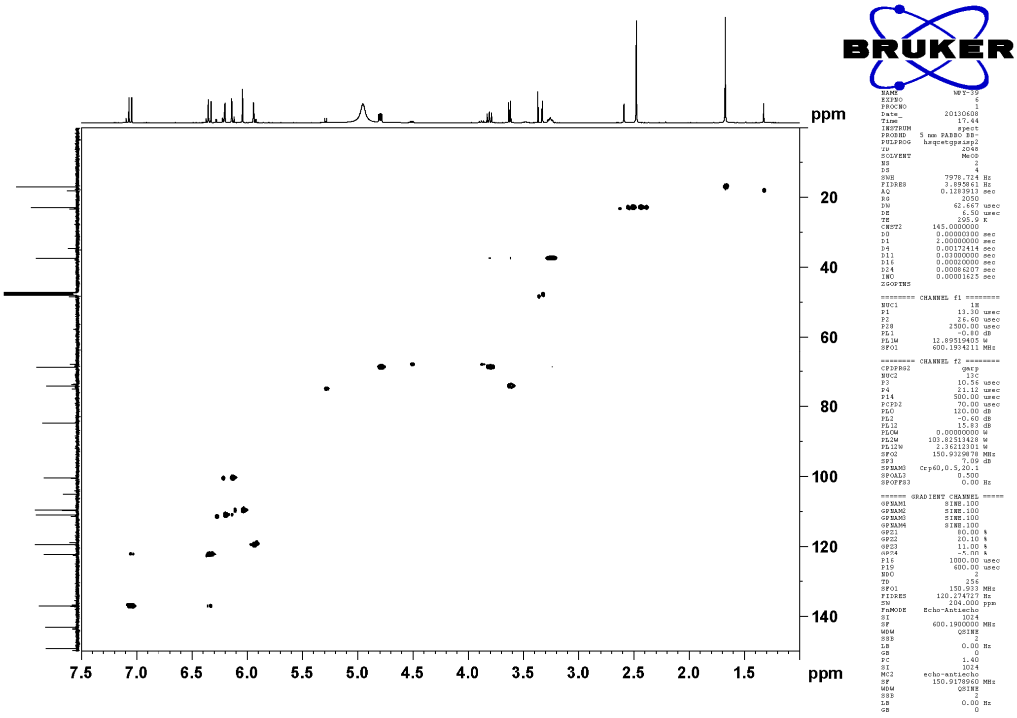

Figure S10. HSQC of **2**.

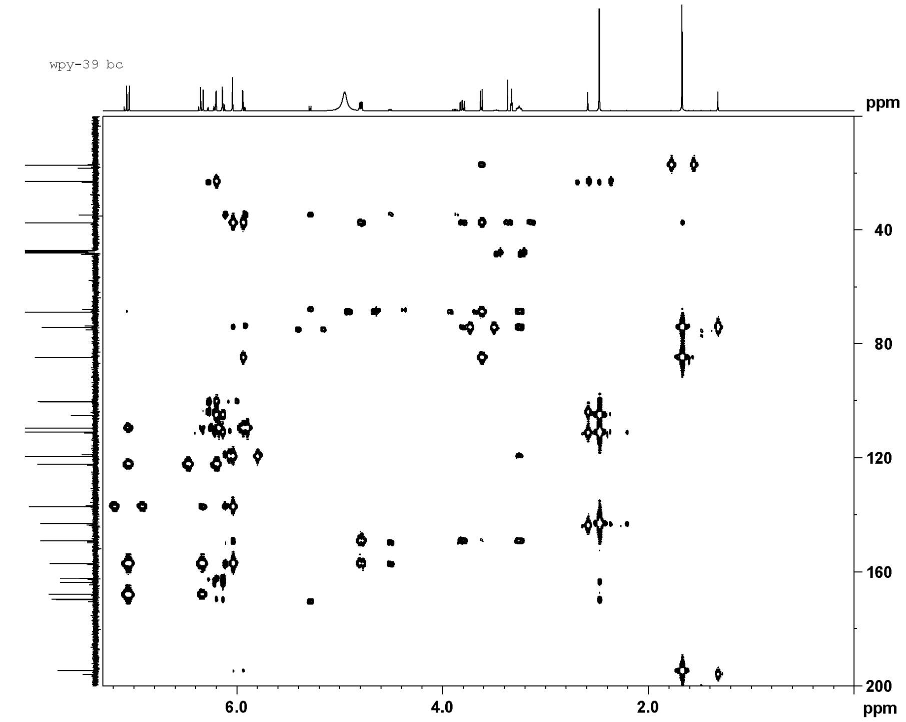

Figure S11. HMBC of **2**.

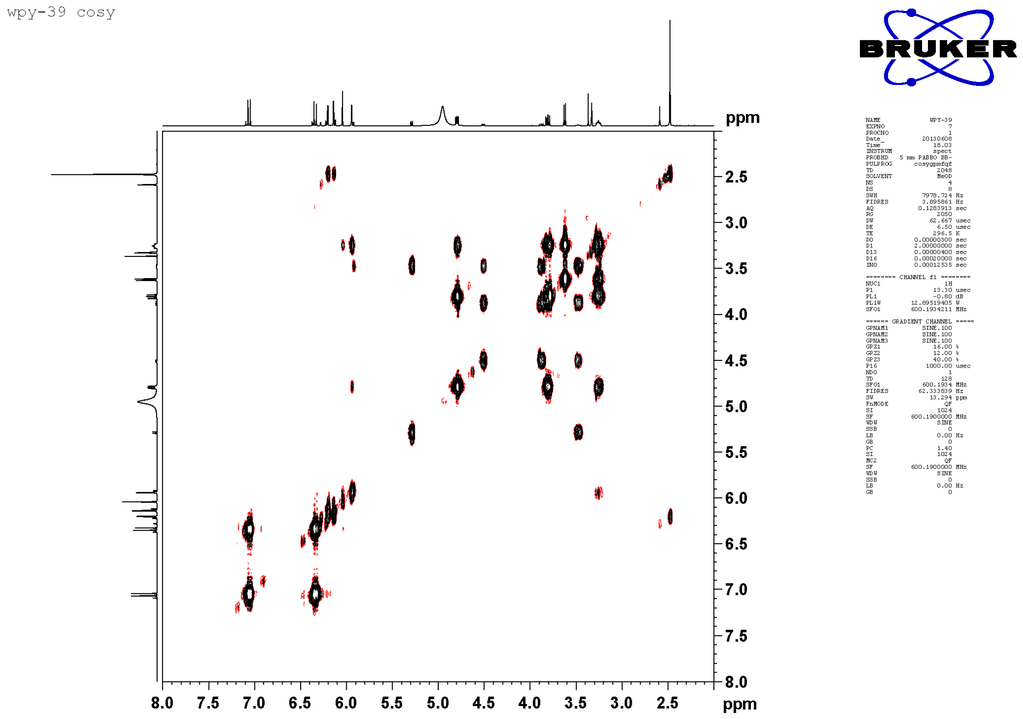

Figure S12. H−H COSY of **2**.

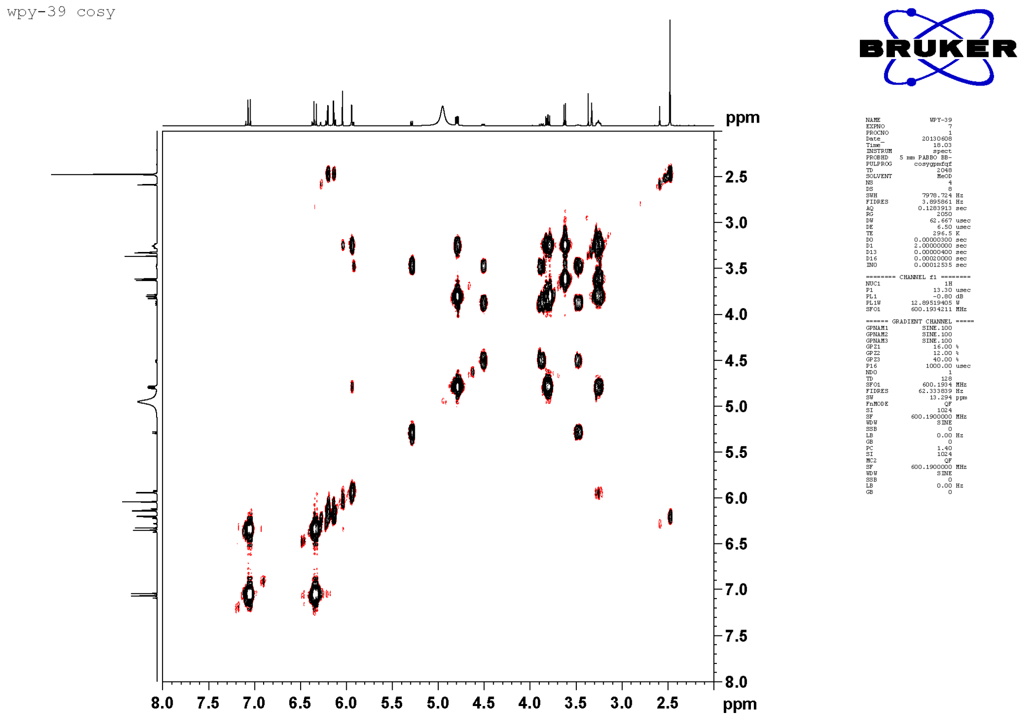

Figure S13. NOESY of **2**.

Mass Spectrum SmartFormula Report

Analysis Info

Analysis Name	D:\Data\wpy-39_000004.d	Acquisition Date	2013-12-8 17:18:54
Method	LCMS_Runing-20131122	Operator	ChuanqiZhou@163.com
Sample Name		Instrument	apex-Ultra

Acquisition Parameter

Polarity	Positive	Source	ESI	No. of Laser Shots	20
Averaged Scans	2	No. of Cell Fills	1	Laser Power	51.0 %
Broadband Low Mass	100.3 m/z	End Plate	3500.0 V	MALDI Plate	300.0 V
Broadband High Mass	1200.0 m/z	Capillary Entrance	4000.0 V	Imaging Spot Diameter	2000.0 µm
Acquisition Mode	Single MS	Skimmer 1	20.0 V		
Pulse Program	basic	Drying Gas Temperature	180.0 °C	Calibration Date	Sun Dec 8 04:39:50 2013
Source Accumulation	0.0 sec	Drying Gas Flow Rate	4.0 L/min	Data Acquisition Size	131072
Ion Accumulation Time	0.1 sec	Nebulizer Gas Flow Rate	1.0 L/min	Apodization	Sine-Bell Multiplication
Flight Time to Acq. Cell	0.0 sec				

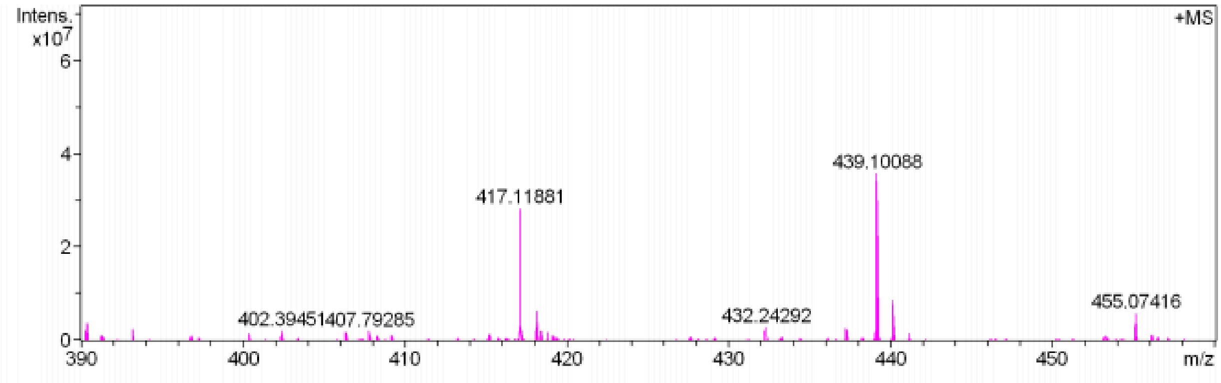

Meas. m/z	#	Formula	Score	m/z	err [mDa]	err [ppm]	mSigma	rdb	e¡¥Conf	N-Rule
417.11881	1	C 21 H 21 O 9	100.00	417.11801	-0.8	-1.9	3.3	11.5	even	ok

Figure S14. Mass Spectrum Smart Formula Report of **2**.

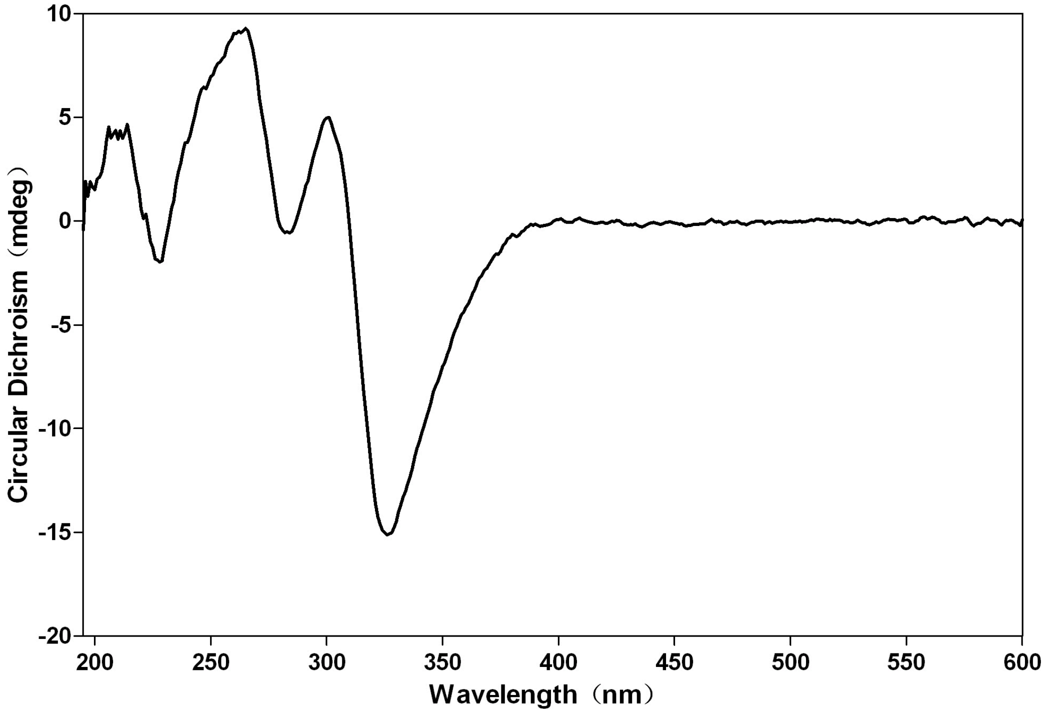

Figure S15. The circular dichroism (CD) spectrum of **2**.

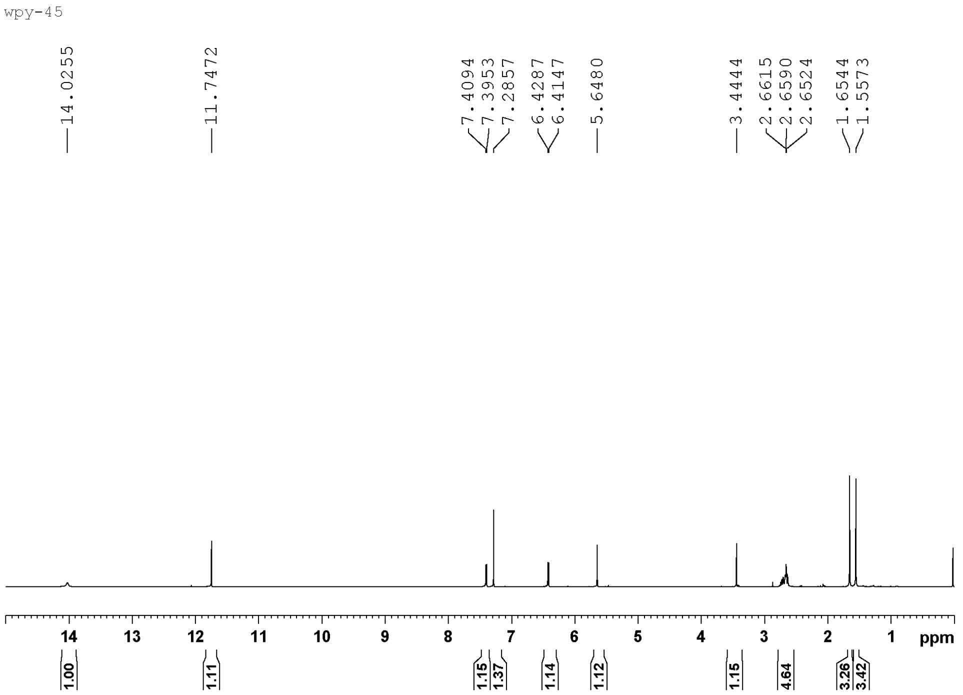

Figure S16. ^{1}H−NMR of **3**.

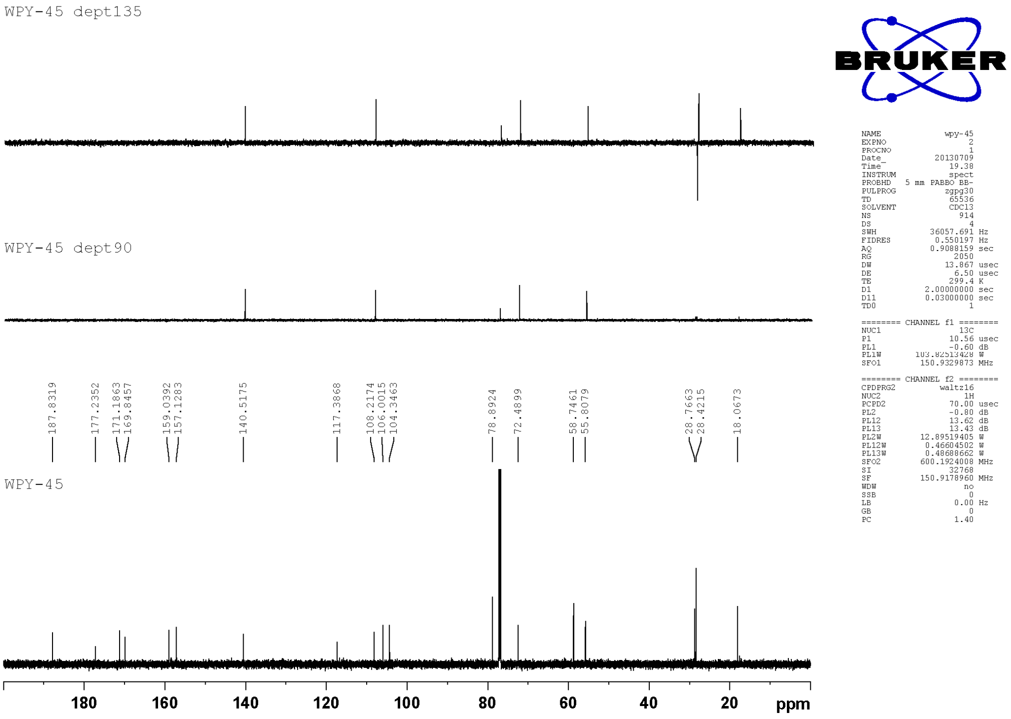

Figure S17. ^{13}C−NMR of **3**.

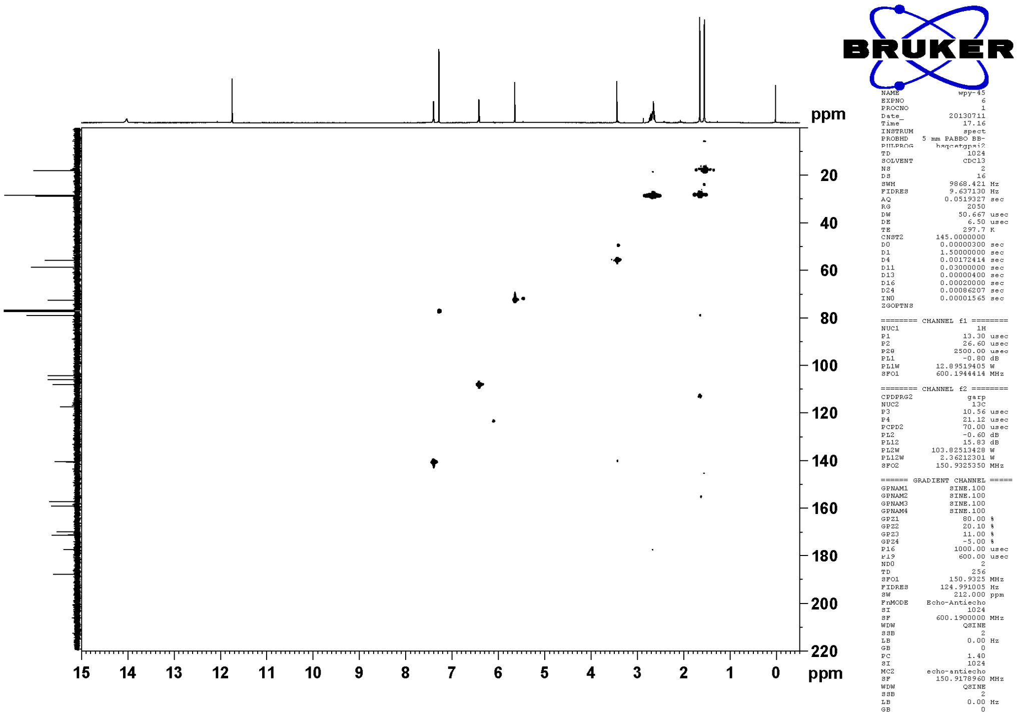

Figure S18. HSQC of 3

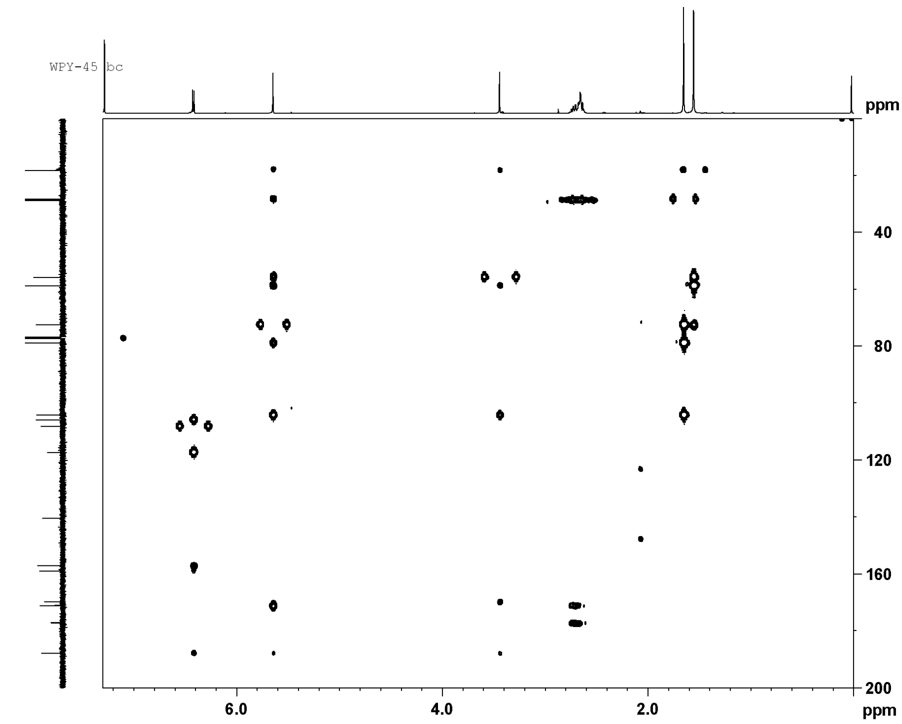

Figure S19. HMBC of **3**.

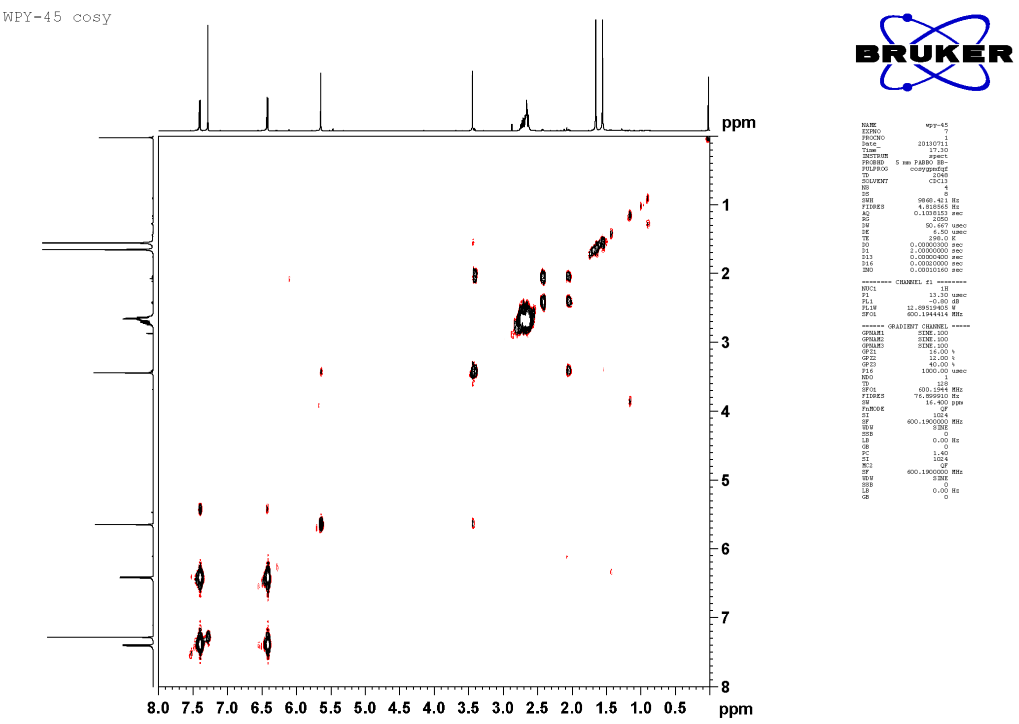

Figure S20. H−H COSYof **3**.

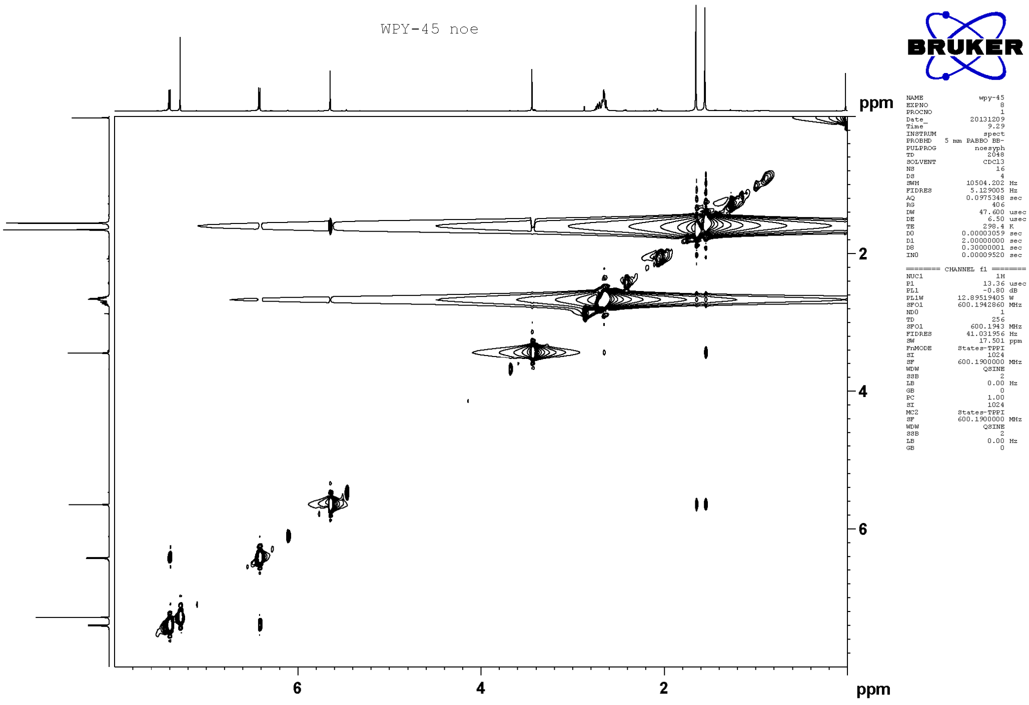

Figure S21. NOESY of **3**.

Elemental Composition Report **Page 1**

Single Mass Analysis
Tolerance = 10.0 PPM / DBE: min = -10.0, max = 120.0
Selected filters: None

Monoisotopic Mass, Odd and Even Electron Ions
22 formula(e) evaluated with 1 results within limits (up to 51 closest results for each mass)
Elements Used:
C: 0-200 H: 0-400 N: 1-1 O: 8-11

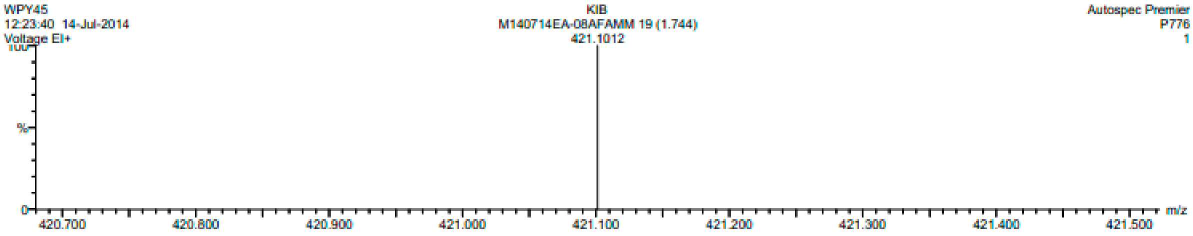

Minimum:				-10.0		
Maximum:		200.0	10.0	120.0		
Mass	Calc. Mass	mDa	PPM	DBE	i-FIT	Formula
421.1012	421.1009	0.3	0.7	11.0	5546025.5	C19 H19 N O10

Figure S22. Single Mass Analysis of **3**.

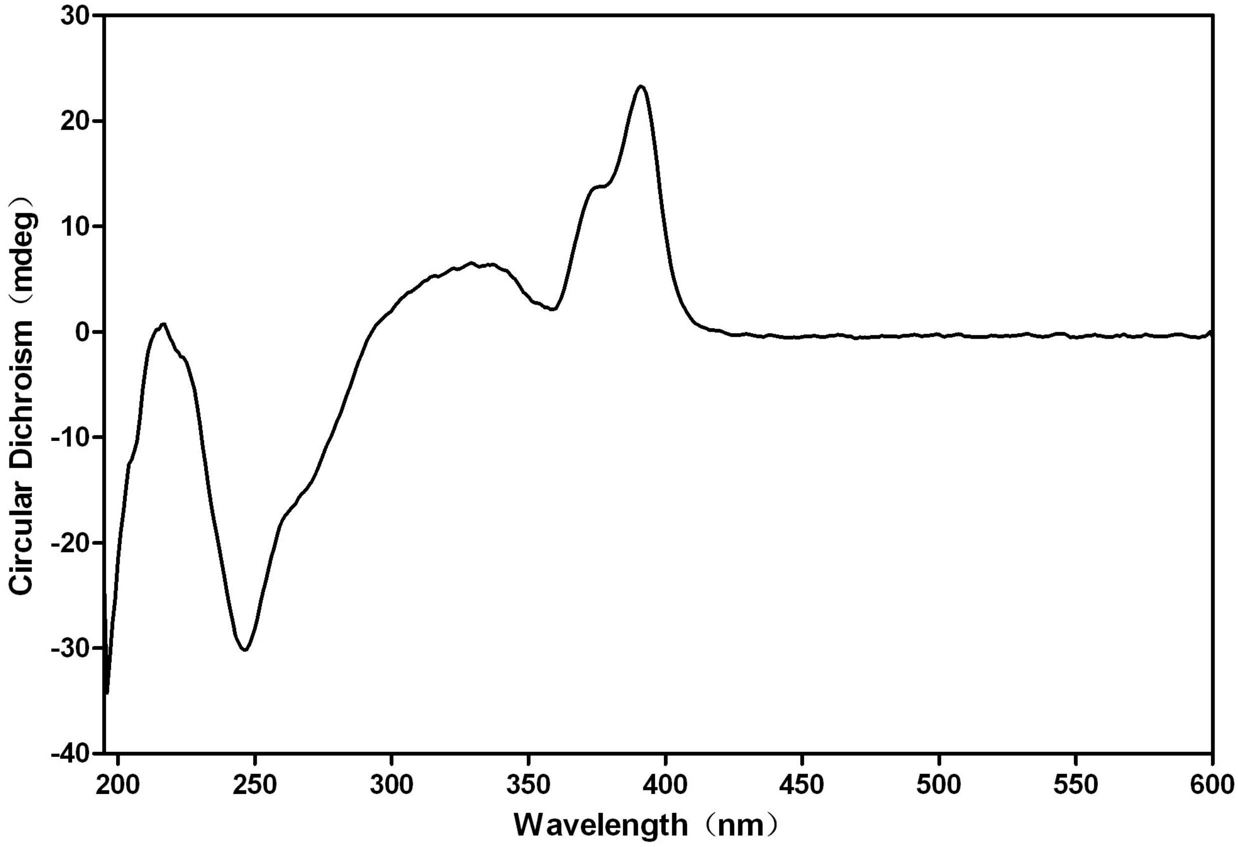

Figure S23. The circular dichroism (CD) spectrum of **3**.
